# Expression and Role of the Calcium-Sensing Receptor in Rat Peripheral Blood Polymorphonuclear Neutrophils

**DOI:** 10.1155/2017/3869561

**Published:** 2017-09-10

**Authors:** Tai-yu Zhai, Bao-hong Cui, Lei Zou, Jing-ya Zeng, Song Gao, Qianyu Zhao, Yuan Wang, Wan-lin Xie, Yi-hua Sun

**Affiliations:** Department of Clinical Laboratory, Harbin Medical University Cancer Hospital, Harbin 150086, China

## Abstract

The calcium-sensing receptors (CaSRs) play an important role in many tissues and organs that are involved in inflammatory reactions. Peripheral blood polymorphonuclear neutrophils (PMNs) are important inflammatory cells. However, the expression and functions of CaSR in peripheral blood PMNs are still not reported. In this study, we collected rat peripheral blood PMNs to observe the relationship between CaSR and PMNs. From the results, we found first that the CaSR protein was expressed in PMNs, and it increased after PMNs were activated with fMLP. In addition, CaSR activator cincalcet promoted the expression of CaSR and P-p65 (NF-*κ*B signaling pathway protein) and Bcl-xl (antiapoptosis protein), and it increased the secretion of interleukin-6 (IL-6) and myeloperoxidase (MPO); meanwhile, it decreased proapoptosis protein Bax expression and the production of IL-10 and reactive oxygen species (ROS). At the same time, cincalcet also decreased the PMN apoptosis rate analyzed by flow cytometry. However, CaSR inhibitor NPS-2143 and NF-*κ*B signaling pathway inhibitor PDTC reverse the results cited earlier. All of these results indicated that CaSR can regulate PMN functions and status to play a role in inflammation, which is probably through the NF-*κ*B signaling pathway.

## 1. Introduction

Peripheral blood polymorphonuclear neutrophils (PMNs) are the main inflammatory cells in the circulatory system. When inflammation occurs, PMNs are activated and recruited into the diseased lesion and they secrete a variety of enzymes, cytokines, or reactive oxygen species (ROS) to stimulate the body's response against many pathogens [1]. Certainly, excessive inflammation reactions can also result in damage. In some inflammation diseases, such as vasculitis, arthritis, and AMI (acute myocardial infarction), PMNs may infiltrate into tissues and are inappropriately activated [2, 3]. So, the PMNs' function should be activated both properly and correctly.

Chèvre et al. suggested that in the atherosclerosis process, PMNs were activated and recruited in the early stage of disease and they caused an inflammatory response by capturing platelets in live mice [4]. In acute lung injury, the continued and uncontrolled inflammatory response results in PMN infiltration into the alveolar and pulmonary edema, thereby damaging the respiratory function. And reducing the quantity and infiltration of activated PMNs was beneficial to acute lung injury [5]. Evidence about PMNs' role in diseases has been mentioned in some research. Carbone et al. have reported that neutrophils infiltrated coronary plaques and the infarcted myocardium and mediated tissue damage by releasing matrix-degrading enzymes and reactive oxygen species. In addition, neutrophils were also involved in adverse cardiac remodeling postinfarction and neointima formation after angioplasty [6, 7]. PMNs can be activated by some factors, including pathogens and *N*-formyl-Met-Leu-Phe (fMLP), PAF, and PMA [8]. These materials can play a part in the PMNs' function by many kinds of pathways; among them, the NF-*κ*B signaling pathway is well known for regulating cell apoptosis and inflammation. Our research group has found that the NF-*κ*B and MAPK signaling pathways were involved in the T lymphocyte apoptosis and the secretion of TNF-*α* and IL-4, all of which were regulated by the calcium-sensing receptor (CaSR) [9].

CaSR belongs to a family C of G protein-coupled receptor (GPCR), and it can maintain calcium homeostasis by sensing extracellular Ca^2+^ concentration changes [10, 11]. The expression and functions of CaSR have been reported in many cells and tissues, including parathyroid gland, gastrointestinal tract, and heart. Lee et al. reported that CaSR could regulate the immunological reaction by controlling the NLR family pyrin domain containing 3 (NLRP3) in macrophages [12]. And, in monocytes, CaSR and G protein-coupled receptors 6A could be activated by extracellular calcium, leading to NLRP3 inflammatory reaction activation [13]. Our group also found that the CaSR in T lymphocytes could influence the AMI onset and progression through the NF-*κ*B signaling pathway [14].

PMNs constitute the most important cellular component in the circulating leucocytes and have a strong killing effect and the highest cytotoxic capacity. An understanding of how to regulate the functions of PMNs and to maintain inflammatory responses at an appropriate level is pivotal for some diseases. However, the expression and functions of CaSR in PMNs and whether CaSR can mediate inflammatory reactions caused by the PMNs are still not reported. In this study, we sought to solve these problems.

## 2. Materials and Methods

### 2.1. Materials

Anti-CaSR-Ab was purchased from NOVUS (Colorado, USA). Anti-P-65-Ab and anti-P-p65-Ab were from Cell Signaling Technology (Boston, USA). Histopaque1083, Histopaque1119, and fMLP were from Sigma (Darmstadt, Germany). NPS-2143, cincalcet, and PDTC were purchased from MCE (New Jersey, USA). Rat IL-6 ELISA kit was from Elabscience (Wuhan, China), rat MPO ELISA kit was from USCN (Wuhan, China), and rat IL-10 was from CUSABIO (Wuhan, China). DHE-ROS kit was from BestBio (Shanghai, China), and NO assay kit was from Beyotime (Shanghai, China).

### 2.2. PMN Isolation

The EDTA-anticoagulated whole blood from adult Wistar rats (200 g–250 g) was collected, and it was then mixed with dextran t-500 (1% *v*/*w*) for 30 min at 37°C. The upper leukocyte-rich layer was transferred to a new tube and centrifuged at 2000*g* for 20 min at 20°C. Pellets were suspended in 2 ml salt solution balanced by D-Hanks; then, they were loaded on the top of the 2 ml Histopaque1119 and Histopaque1083 density gradient carefully and centrifuged at 700*g* for 30 min at 20°C. The cells were collected in the PMN-rich layer between Histopaque1083 and Histopaque1119 and were suspended in D-Hanks. The viability of the cells was determined by Trypan blue dye exclusion. The purity of isolated PMNs was detected by Wright Giemsa staining. PMNs' viability and purity were more than 95%.

### 2.3. Western Blotting Analysis

The PMNs were collected and lysed on ice with protein lysate containing phenylmethylsulphonyl fluoride (PMSF) and phosphatase STOP for 40 min. The protein concentration was determined by using the Brad-ford protein assay with BSA as standard. Total proteins (20 *μ*g) were subjected to 10% SDS-PAGE and blotted onto polyvinylidene fluoride membrane at 20 V for 30 min. After being blocked in TBS-T containing 5% (*w*/*v*) skimmed milk at 37°C for 1 h, the membranes were then incubated overnight at 4°C with various antibody: anti-CaSR (1 : 300), anti-Bcl-xl (1 : 1000), anti-Bax (1 : 1000), anti-p65 (1 : 1000), and anti-P-p65(1 : 1000) respectively, and then incubated with anti-IgG antibody conjugated with horseradish peroxidase diluted 1 : 5000 in TBS-T for 1 h at room temperature. Immune complexes were detected with goat anti-rabbit or rabbit anti-mouse IgG combined with HRP. HRP activity was detected by ECL reagent. Quantitative comparisons of different proteins under different conditions were performed using image J.

### 2.4. Cytokines and MPO Analysis by ELISA

The cytokines and myeloperoxidase (MPO) levels in the medium of cultured PMNs were tested by ELISA kit according to the manufacturer's instructions.

### 2.5. Apoptosis Detection by Flow Cytometry

The apoptotic ratio was measured by flow cytometry as previous described [13]. The D-Hanks-washed PMNs were incubated with 5 *μ*l Annexin V-fluorescein isothiocyanate (FITC) for 15 min at room temperature in dark, and then, 5 *μ*l propidium iodide (PI) staining was added. And, flow cytometry (BD LSRF Ortessa, USA) was used to analyze the apoptosis.

### 2.6. Reactive Oxygen Species Assay

PMNs were stimulated by fMLP and cultured with CaSR agonist cinacalcet, CaSR inhibitor NPS-2143, and NF-*κ*B pathway blocker PDTC at 37°C for 30 min. Then, supernatant was collected and was dyed with dihydroethidium (DHE) at 1 : 100. The fluorescent intensity was viewed by fluorescence microscopy, and the fluorescent concentration was measured by a fluorescence microplate reader.

### 2.7. NO Production Analysis

NO production in PMNs was assessed using NO assay kit following the manufacturer's instructions. Briefly, sodium nitrite (NaNO2) was used as a standard curve. 50 *μ*l supernatant of cultured PMNs was mixed with an equal volume of Griess reagent (1% sulfanilamide, 0.1% naphthylethylenediamine dihydrochloride, and 2.5% phosphoric acid) and incubated at room temperature for 5 min. The concentration of nitrite was measured by reading at 570 nm absorbance wavelength [15].

### 2.8. Data Statistical Analysis

All the data replicated at least three times for each group. The values are presented as mean with ±SEM. SNK post test was performed. Statistical significance among group means was assessed by ANOVA. Differences were considered significant at *p* < 0.05.

## 3. Results

### 3.1. CaSR Protein Was Expressed in PMNs

**Figure 1:**
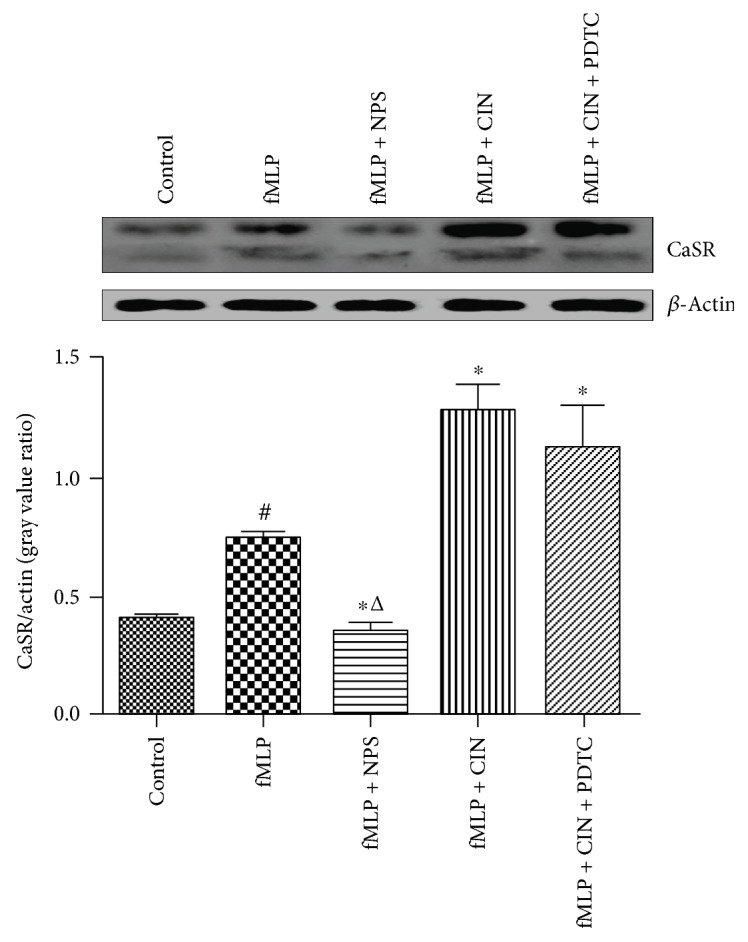
Expression of CaSR proteins in PMNs. We used fMLP (10 nM) to stimulate the cells for 2 min, and the PMNs without fMLP served as the control. CaSR inhibitor NPS-2143 (100 nM), CaSR agonist cinacalcet (100 nM), and NF-*κ*B inhibitor PDTC (100 nM) were added and incubated at 37°C for 30 min. Protein expression results were representative of three experiments. Expression protein was quantified by the gray value. ^#^*P* < 0.05 versus control group, ^∗^*P* < 0.05 versus fMLP group, ^△^*P* < 0.01 versus fMLP + CIN group.

The CaSR protein with two relative molecular masses of 150 kDa and 130 kDa was detected by using Western blotting in PMNs. The results showed that the CaSR protein was expressed in PMNs, and its expression was increased after PMNs were stimulated by fMLP, which is a kind of inflammation activator. And, CaSR agonist cinacalcet significantly increased the expression of CaSR on activation of PMNs, whereas CaSR inhibitor NPS-2143 effectively decreased the expression of CaSR. The NF-*κ*B pathway inhibitor PDTC exhibited no obvious change (Figure 1).

### 3.2. Detection of Bcl-xl and Bax Expression

**Figure 2:**
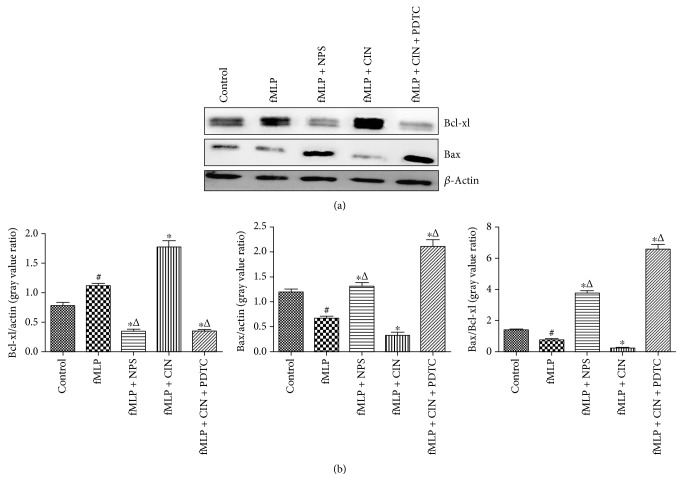
The expressions of Bcl-xl and Bax in PMNs detected by WB quantitative analysis. PMNs were treated with f-MLP (10 nM), NPS-2143 (100 nM), cinacalcet (100 nM), and PDTC (100 nM) for 30 min. Protein expression was quantified by the gray value (a). Results were quantified by densitometry (b). Expression results are representative of three experiments. ^#^*P* < 0.05 versus control group, ^∗^*P* < 0.05 versus fMLP group, and ^△^*P* < 0.01 versus fMLP + CIN group.

Next, we wanted to observe the role of CaSR in the PMNs. Bcl-xl is an antiapoptotic protein, and Bax is a proapoptotic protein. From the results, it can be found that Bcl-xl protein was increased after PMNs were stimulated by fMLP, and CaSR agonist cinacalcet further increased the expression. However, NPS-2143 and PDTC inhibited Bcl-xl protein expression effectively. Meanwhile, the opposite results were obtained about Bax protein expression with the same treatment (Figure 2).

### 3.3. PMNs' Apoptosis Rate Analysis

**Figure 3:**
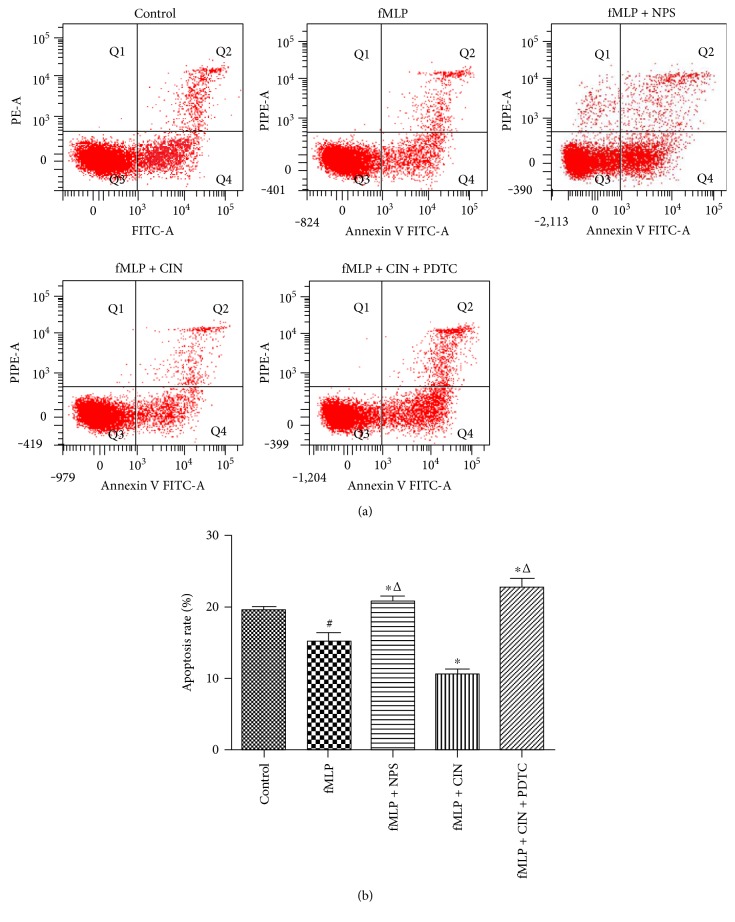
Apoptosis rate of PMNs was detected by flow cytometry (*n* = 9). We used fMLP (10 nM) to stimulate PMNs for 2 min, the cells without fMLP as control. CaSR inhibitor NPS-2143 (100 nM), CaSR agonist cinacalcet (100 nM), and NF-*κ*B inhibitor PDTC (100 nM) were added in cultured PMNs at 37°C for 30 min, and PMNs' apoptosis rate was detected. Results were representative of three experiments (a). The apoptosis rate under different conditions was quantified by densitometry (b). ^#^*P* < 0.05 versus control group, ^∗^*P* < 0.05 versus fMLP group, and ^△^*P* < 0.01 versus fMLP + CIN group.

PMNs' lifespan is short; they are sacrificed soon after battling with pathogens. In vitro, PMNs' apoptosis also occurs in a short time. However, we found that fMLP reduced the apoptosis rate, and CaSR activation further decreased the apoptosis rate. On the contrary, NPS2143 and PDTC increased the apoptosis rate. These indicated that CaSR can regulate PMNs' survival state (Figure 3).

### 3.4. Production of Cytokines

**Figure 4:**
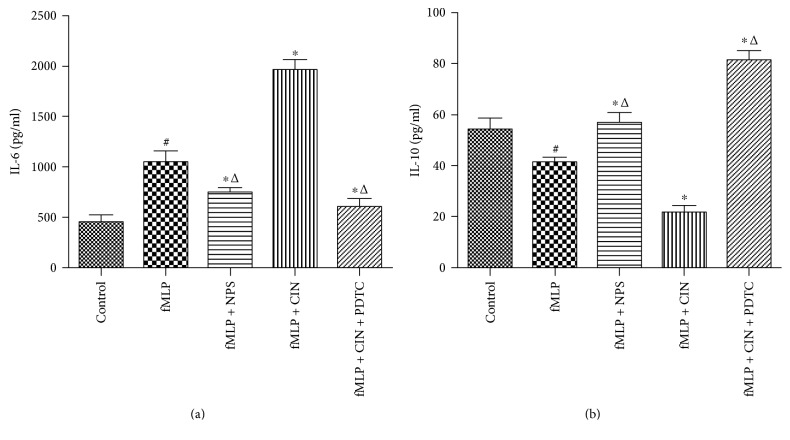
The levels of cytokines in the different groups (*n* = 9). The production of IL-6 (a) and IL-10 (b) in supernatant of cultured PMNs was detected by ELISA. PMNs were cultured with NPS-2143 (100 nM), cinacalcet (100 nM), and PDTC (100 nM) for 12 h after stimulated by fMLP (10 nM) for 2 min. ^#^*P* < 0.05 versus control group, ^∗^*P* < 0.05 versus fMLP group, and ^△^*P* < 0.01 versus fMLP + CIN group.

PMNs are involved in inflammatory responses in a variety of ways, such as secretion of cytokines, adhesion of endothelial cells, and recruitment of other immunological cells. Among them, cytokines exhibit direct and strong effects. In our study, we found that production of proinflammatory cytokine IL-6 was increased in the fMLP group, and the CaSR activator further enhanced IL-6 secretion. And, the IL-6 concentration was reduced when CaSR activation was inhibited. PDTC produced the same effect as the NPS2134. On the other hand, the change trend of anti-inflammatory cytokine IL-10 was opposite with IL-6 under the same conditions (Figure 4).

### 3.5. Production of ROS and MPO

**Figure 5:**
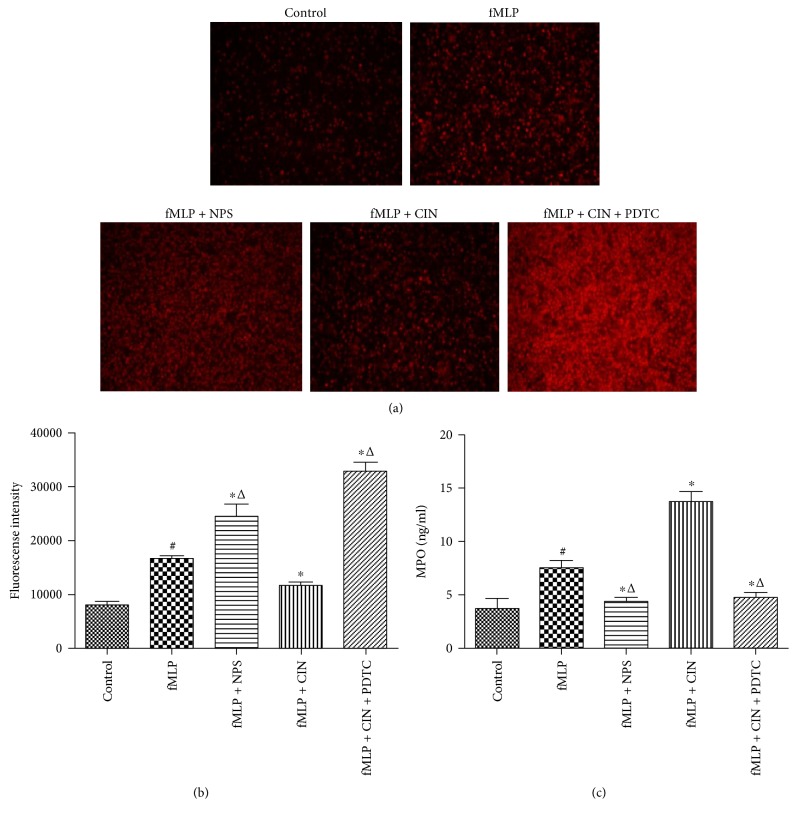
The production of ROS and MPO in PMNs (*n* = 9). ROS were dyed with DHE probe at 1 : 100. And, the fluorescent intensity was viewed by fluorescence microscopy at the excitation wavelength 535 nm and emission wavelength 610 nm (a), and the ROS content was measured by a fluorescence microplate reader (b). The production of MPO in the supernatant of cultured PMNs was detected by ELISA (c). ^#^*P* < 0.05 versus control group, ^∗^*P* < 0.05 versus fMLP group, and ^△^*P* < 0.01 versus fMLP + CIN group.

PMNs can produce a series of reactive oxygen species (ROS) that play a role during metabolic processes. In our research, a fluorescent probe was utilized to dye intracellular ROS in PMNs. Results showed that the fluorescence intensity in PMNs activated by fMLP increased slightly. NPS2134 and PDTC made the intensity brighter. However, cinacalcet darkened the fluorescence brightness. These changes were consistent with the intracellular ROS concentration in PMNs measured by the fluorescence microplate reader (Figures 5(a), and 5(b)).

MPO is a PMN marker whose level and activity change represent the function and activity of PMN. MPO produces and regulates the inflammatory response in many aspects. In this study, MPO was detected by ELISA. The MPO production was increased when PMNs were activated by fMLP, and CaSR activator further enhanced MPO production, which was reduced when CaSR activation was inhibited (Figure 5(c)).

### 3.6. NO Production Measurement

**Figure 6:**
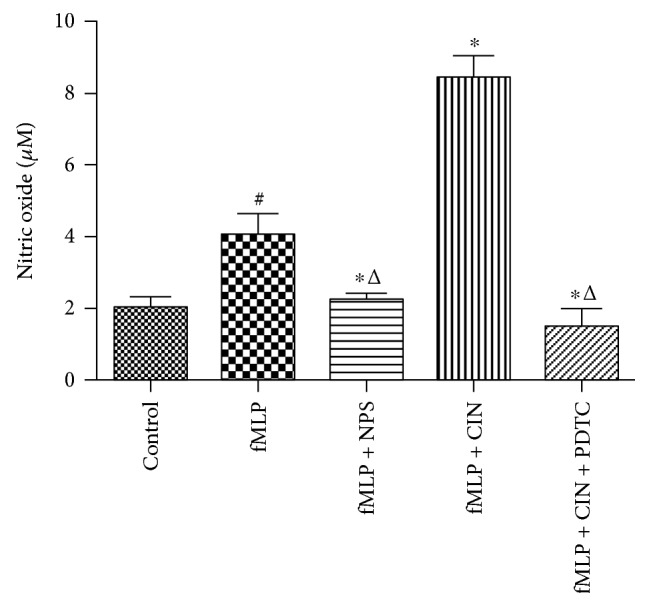
The level of NO in the different groups (*n* = 9). PMNs were treated with NPS-2143 (100 nM), cinacalcet (100 nM), and PDTC (100 nM) for 12 h after stimulated by fMLP (10 nM) for 2 min. The production of NO in supernatant of cultured PMNs was detected. ^#^*P* < 0.05 versus control group, ^∗^*P* < 0.05 versus fMLP group, and ^△^*P* < 0.01 versus fMLP + CIN group.

NO also plays a key role in the process of neutrophil functions. In our experiment, NO concentration increased in the supernatant of cultured PMNs after neutrophils were activated by fMLP and the CaSR agonist could further increase the yield of NO, but this effect was inhibited by NPS-2143 and PDTC (Figure 6).

### 3.7. Phosphorylation of p65 in PMNs

**Figure 7:**
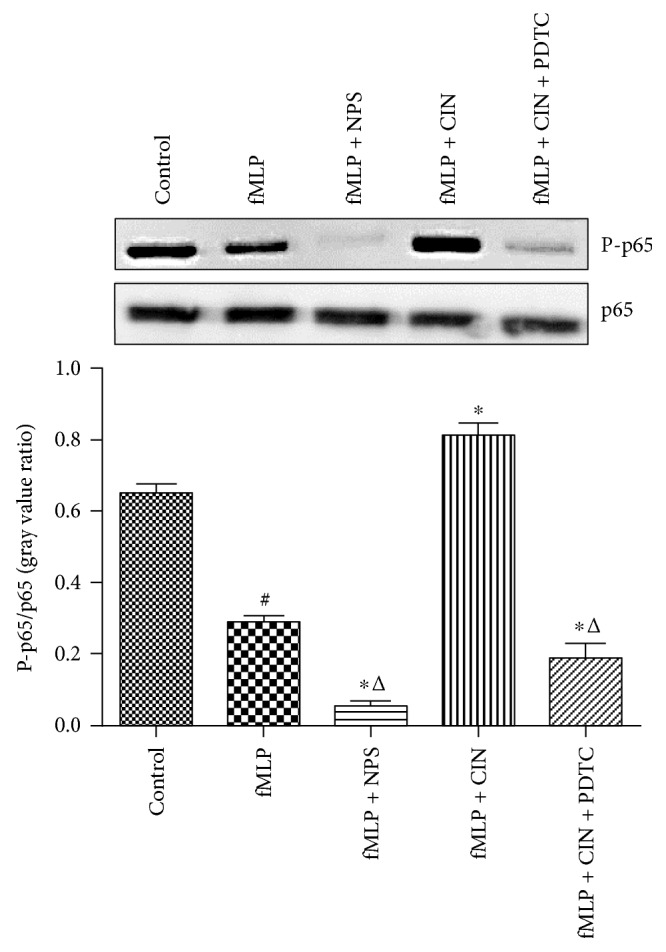
The expressions of P-p65 and p65 in PMNs (*n* = 9). PMNs were treated with NPS-2143 (100 nM), cinacalcet (100 nM), and PDTC (100 nM) for 30 min after PMNs were stimulated with fMLP (10 nM). Expression protein was quantified by the gray value. Expression results are representative of three experiments. ^#^*P* < 0.05 versus control group, ^∗^*P* < 0.05 versus fMLP group, and ^△^*P* < 0.01 versus fMLP + CIN group.

The NF-*κ*B pathway is an important signaling transduction pathway in some physiological or pathological events. p65 is the key protein in the NF-*κ*B pathway. In our experiment, we identified that the phosphorylation of p65 decreased in stimulated PMNs. But, cinacalcet significantly increased the p65 phosphorylation level, and NPS-2143 and PDTC markedly reduced its expression (Figure 7).

## 4. Discussion

The expression and functions of CaSR were reported in many kinds of blood cells, such as lymphocytes and monocytes. In this study, we proved first the expression of CaSR in PMNs and also found that the expression of CaSR protein increased after PMNs were activated.

CaSRs play an important role in regulating Ca^2+^ homeostasis. Our team has researched the role of CaSR in cardiomyocytes and T lymphocytes. CaSR can induce cardiomyocyte apoptosis in AMI and enhance T cells to secrete some kinds of cytokines in sepsis [9, 14]. Then, what role does the CaSR play in PMN? In this study, we found that Bax protein and apoptosis rate of activated PMNs slightly decreased and a significant decrease was triggered by the CaSR activator. However, the CaSR inhibitor increased the apoptosis. On the other hand, the antiapoptotic protein Bcl-xl had opposite changes compared with Bax. These results indicated that CaSR activation can reduce PMN apoptosis. And, we speculated that CaSR enabled the PMN apoptosis to be delayed and to have a longer time to combat with the pathogen in the inflammation region.

We all know that PMNs had a high value in the inflammation reaction. They can be recruited in diseased tissue and secrete a variety of inflammatory factors and chemokines that participate in the development of the disease. Harbort et al. reported that neutrophils played an essential role in the initial stages of inflammation by balancing pro- and anti-inflammatory signals. Among these signals were the production of proinflammatory cytokines and the timely initiation of anti-inflammatory cell death via constitutive apoptosis. It was also found that neutrophils isolated from patients with ataxia-telangiectasia (AT) overproduced proinflammatory cytokines and had a prolonged lifespan compared with healthy controls. This effect was partly mediated by increases in activation of p38 MAP kinase [16]. So, we all agree that cytokines are powerful weapons of PMN. IL-6 is a proinflammation factor, and IL-10 is an anti-inflammation factor. In this study, we observed that CaSR activation induced the IL-6 production increase and IL-10 secretion decrease. This means that CaSR promoted PMNs to secrete more proinflammation factors to strengthen the inflammation reaction.

Many studies supported that IL-6 increased apoptosis. In our study, we also found that CaSR activation promoted PMNs to secrete more proinflammation factors IL-6. But, our final conclusion was CaSR activation can reduce PMN apoptosis. How to explain this? There are also some researches that show IL-6 can enhance the expression of some antiapoptotic proteins, such as MCL-1 and BCL-XL, thereby promoting the antiapoptotic effect of peripheral blood neutrophils in patients [17]. Wang et al. suggested that IL-6 can downregulate the expression of BAX and caspase-3 activity to inhibit the apoptosis of PMNs and prolong the survival time of PMNs, and IL-6 neutralizing antibody increased the rapid apoptosis of PMNs [18]. On the other hand, we also found that CaSR agonist activated CaSR/NF-*κ*B/BCL-XL pathway to decrease PMN apoptosis. From the results, we can conclude that CaSR/NF-*κ*B/BCL-XL pathway may be leading and overwhelming compared with IL-6 role in regulating PMN apoptosis.

MPO is a key enzyme and a marker about the activity of PMNs. MPO is abundantly present in neutrophils, and the proportion of MPO in neutrophils can reach more than 5%. Most of MPO, approximately 95%, are produced by PMNs [19, 20]. MPO can oxidize low-density lipoprotein and extracellular matrix, resulting in endothelial cell damage in the blood vessel [21]. Reber et al. reported that neutrophils can contribute to optimal host protection by limiting the extent of endotoxin-induced inflammation in an MPO-dependent manner [22]. We obtained the results that CaSR activation can increase the MPO concentration in the supernatant of the cultured PMNs and the CaSR inhibitor decreased the content. This suggested that CaSR activation stimulated the PMNs' activity.

Reactive oxygen species (ROS) are generated during mitochondrial oxidative metabolism as well as in cellular response to xenobiotics, cytokines, and bacterial invasion. ROS is an important effector in inflammation. Some evidence indicated that ROS also serve as critical signaling molecules in cell proliferation and survival. Oxidative stress resulted in direct or indirect ROS-mediated damage of nucleic acids, proteins, and lipids, and it had been implicated in carcinogenesis, neurodegeneration, atherosclerosis, diabetes, and aging. ROS have also been shown to promote tumor metastasis through gene activation [23]. Neutrophil extracellular traps (NETs) have been associated with cancer metastasis and cancer-associated thrombosis, but the release of NETs has been shown to be requiring reactive oxygen species (ROS) production [24] And, Pillay et al. suggested that PMN-derived ROS may repress the activation and proliferation of T lymphocytes [25]. So, we presumed that CaSR may be beneficial for regulating the degree of inflammatory injury combined with other immune cells. Our results showed that intracellular ROS concentration was slightly increased after PMNs were stimulated by fMLP. However, the CaSR activator inhibited the intracellular ROS concentration in PMN and it also increased the production. This result was contradictory with MPO change. Theoretically, increased MPO should trigger more ROS production [26]; on the contrary, here, increased MPO resulted in decreased ROS. We speculated that this may have been due to not enough motivation of intense pathogens in the environment. And, the role of CaSR in inhibiting the production of ROS in PMNs was dominant.

In addition, our experiment showed that CaSR activation stimulated PMNs to produce more NO. NO could influence chemotaxis, adhesion, and apoptosis of PMNs in different pathophysiological conditions [27]. NO-mediated production of ROS in PMNs has been reported; however, the role of NO is biphasic on the production of ROS in PMNs after treatment with ascorbate, arachidonic acid, and fMLP. The production of ROS was increased in lower NO concentrations, whereas higher NO concentrations inhibited ROS production through the inhibition of NADPH oxidase or ROS clearaway [28]. So, we presumed that ROS production induced by CaSR seemed to be more closely related to NO. Moreover, some studies [29] have suggested that BAX activation can lead to an increase in ROS production, which is consistent with our results. In our results, CaSR activation decreased Bax expression, which was accompanied by the decrease in ROS production. It was also proved that CaSR activation can lessen PMNs' damage to the surrounding tissues by reducing ROS production.

CaSR can activate multiple cell signaling pathways, which were involved in many different physiological functions, including apoptosis and proliferation. In our previous study, we demonstrated that CaSR protein in T lymphocytes regulated the production of cytokines and apoptosis through the NF-*κ*B signaling pathway in sepsis [9, 30]. Activation of the NF-*κ*B pathway can lead to the production of multiple inflammatory factors [31]. In this study, we found that the NF-*κ*B pathway blocker PDTC decreased P65 phosphorylation, and it increased the expression of Bcl-xl and the secretion of IL-6 and MPO; meanwhile, it increased PMNs' apoptosis rate and the expression of Bax and IL-10 and ROS production. These item changes were similar to the CaSR inhibitor NPS 2134. Overall, we concluded from all these results that the role of CaSR in PMNs has a link with the NF-*κ*B pathway.

In our experiments, there was a paradoxical phenomenon: The expression of CaSR was increased after PMNs were treated by fMLP, whereas the activity of p-P65 was mildly inhibited. It has been reported that ROS have a two-way regulation role in the NF-*κ*B pathway; that is, a low concentration of ROS can activate the NF-*κ*B pathway protein, and a high concentration inhibits its activity [32]. So, we speculated that our results may be due to the different concentration of ROS.

Both of the activity and expression quantity of CaSR have changed induced by CaSR agonist or inhibitor. Which was more important to PMNs' function? From the results, we found that fMLP increased the CaSR expression 2.04 times compared to control (fMLP\control) and fMLP + CIN increased about 1.84 times compared to fMLP (fMLP + CIN\fMLP). Then, we observed that fMLP\control increased BcL-xl expression 1.12 times compared with fMLP + CIN\fMLP 1.56 times, increased Il-6 production 1.86 times compared with fMLP + CIN\fMLP 2.06 times, and increased MPO production 1.27 times compared with fMLP + CIN\fMLP 2.18 times. From these data, we can conclude that the activity of CaSR plays a more important role in regulating PMNs' function.

All these obtained results suggested that CaSR in PMNs was striving toward reducing apoptosis and ROS production to diminish the damage caused by PMNs in spite of promoting the proinflammation factor, which was associated with the NF-*κ*B pathway. This provides a basis for further study of the function of PMNs, as well as the rational regulation of the function of neutrophils to prevent inflammatory damage in the course of various inflammatory diseases. Certainly, our test results need to be further validated in animal experiments.
